# Case Report on Mycobacterium tuberculosis Presenting As Lemierre’s Syndrome: A Reemerging Catastrophe

**DOI:** 10.7759/cureus.56353

**Published:** 2024-03-17

**Authors:** Pranav Chaudhari, Rucha Sawant, Gautam N Bedi, Rahul Desale, Sunil Kumar, Sourya Acharya

**Affiliations:** 1 Medicine, Jawaharlal Nehru Medical College, Wardha, IND; 2 Interventional Radiology, Jawaharlal Nehru Medical College, Wardha, IND

**Keywords:** role of mri, mycobacterium tuberculosis (mtb), obstructive hydrocephalus, pulmonary tuberculosis, tubercular meningitis, lemirre’s syndrome

## Abstract

Lemierre's syndrome is characterized by internal jugular vein thrombophlebitis and bacteremia, primarily from anaerobic organisms. The condition usually arises after a recent oropharyngeal infection. Young, healthy people with prolonged pharyngitis that progresses into septicemia, pneumonia, or lateral neck stiffness should be suspected of having Lemierre's syndrome. Identifying internal jugular vein thrombophlebitis and developing anaerobic bacterial growth on blood culture are frequently used to confirm the diagnosis. Treatment consists of long-term antibiotic treatment, sometimes in conjunction with anticoagulant medication.

In this case report, we describe the unique case of a 29-year-old male with *Mycobacterium tuberculosis* with pulmonary tuberculosis, tubercular meningitis, tuberculosis-related acute ischemic stroke with septic thrombophlebitis. The patient presented with sudden onset altered sensorium for 4 hours. Magnetic resonance imaging of the brain was done, which suggested obstructive hydrocephalus with periventricular ooze. The patient was started on antibacillary treatment, antibiotics, anticoagulants, and systemic steroids. The patient was vitally stable when he was discharged. Therefore, it is crucial to consider the likelihood of such atypical tuberculosis presentations while providing a prompt and relevant diagnosis and recommending the right course of therapy.

## Introduction

Lemierre’s syndrome, also known as necrobacillosis or post-anginal septicemia, is characterized by bacteremia, metastatic septic emboli secondary to acute pharyngeal infections, and internal jugular vein (IJV) thrombophlebitis. There is still debate regarding the exact meaning of Lemierre's syndrome. *Fusobacterium necrophorum* is typically to blame since it can cause embolic abscesses and superior IJV thrombosis. Nonetheless, the condition may also be considered in cases of anaerobic septicemia resulting from several infection sources, including the intestinal tract, the genitourinary tract, and the upper respiratory tract [1].

One of the most severe manifestations of tuberculosis (TB), which accounts for over 40% of tuberculosis fatalities in developing nations and accounts for 10% of all TB cases, is tuberculous meningitis (TBM) [2]. The development of tuberculoma, hydrocephalus, and cerebral stroke are the three primary adverse outcomes of TBM [3]. However, there was a lack of knowledge regarding the stroke risk factors in TBM patients, particularly in young individuals. There have been documented cases of cerebral tubercular thrombophlebitis [4]; we suspect similar mechanisms causing IJV thrombophlebitis in our case.

The association of pulmonary tuberculosis with the IJV is unique. Hence, we report the case of a young male along with a relevant literature review. TB is a global health concern, and through this case report, we want to emphasize the importance of suspecting Lemierre’s syndrome in such cases.

## Case presentation

A 29-year-old male with complaints of sudden onset weakness of bilateral lower limbs, vomiting, and dizziness followed by altered sensorium presented to the casualty. The patient initially had complaints of low-grade fever for 15 days, dizziness, and generalized weakness, for which he took treatment at a local hospital on an outpatient basis. The patient has been a chronic alcoholic for the last 12 years with a daily intake of 90-120 ml country liquor, with the previous intake being seven days back. The fever was low grade, associated with evening rise, and not relieved on anti-pyretic medications. The weakness progressed over

10 days, and the patient required help to perform day-to-day activities (Table 1). At the time of presentation, the patient’s father gave a history of dizziness followed by sudden onset loss of consciousness and altered sensorium. Taking into account the low score on the Glasgow coma scale (GCS), tachycardia, and tachypnoea, the patient was intubated and put on a mechanical ventilator.

**Table 1:** Timeline of history

Days prior to admission	Event
15	Low-grade evening rise fever
12	Generalised weakness
7	Support to walk
6	Inability to do day-to-day activities
4	Severe cough associated with expectoration
2	Dizziness
0	Loss of consciousness

The patient arrived at the casualty department unconscious, with a low-grade fever of 100 Fahrenheit, tachycardia with a heart rate of 130 per minute, and tachypnea with a respiration rate of 33 cycles. He was in severe distress with air entry severely diminished in bilateral lungs with mild basal crepitations heard on auscultation in both lungs. Central nervous system examination suggested a poor Glasgow coma scale score of (GCS) E2V1M1 with bilateral plantar extensor and bilateral pupil mid-dilated reactive to light.

**Figure 1:**
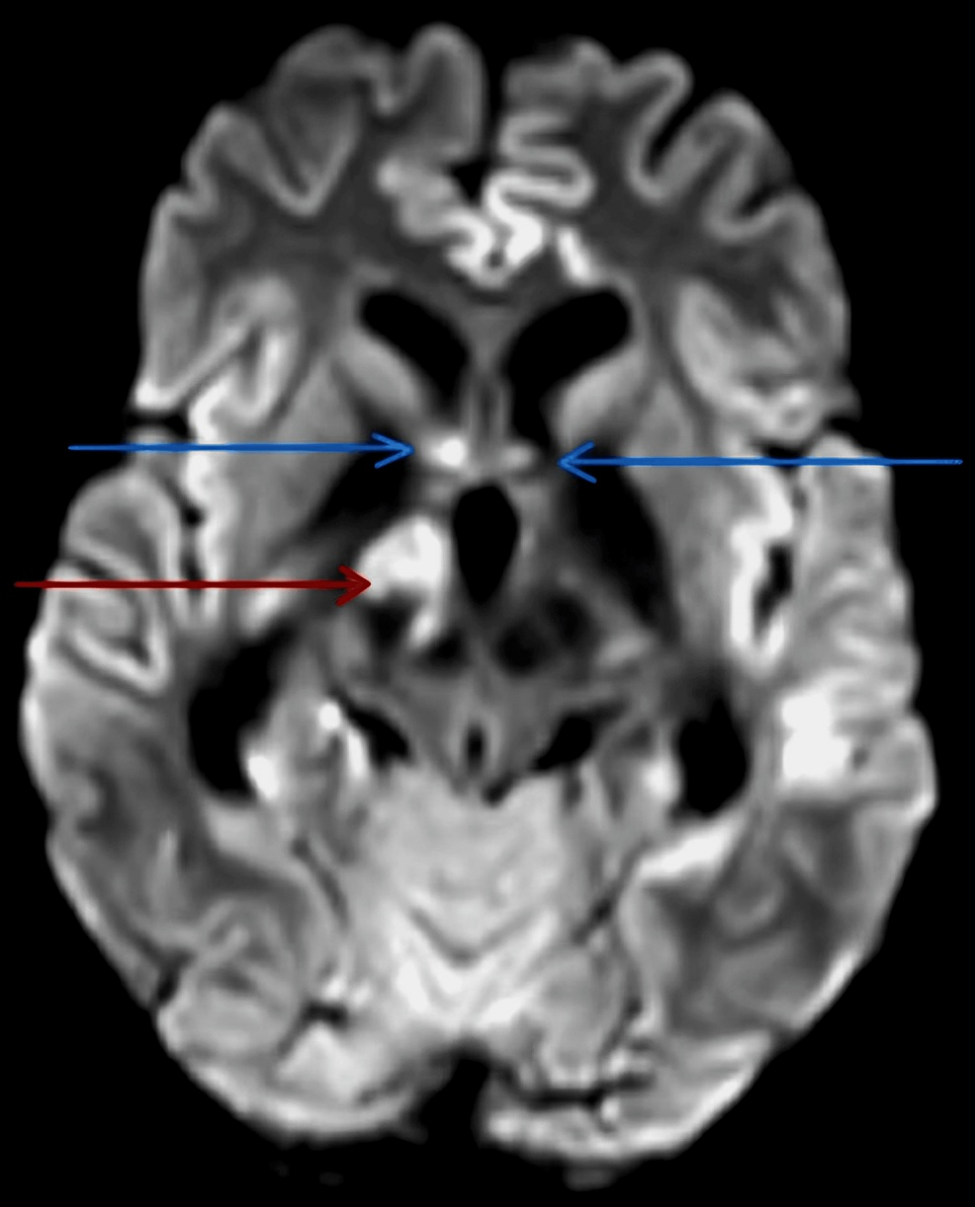
MRI brain showing areas of restricted diffusion in the right thalamus (red arrow) and bilateral basi-frontal region, adjacent to bilateral temporal horns of lateral ventricles (blue arrows). MRI- Magnetic Resonance Imaging

In view of the altered sensorium, a contrast-enhanced MRI brain was done for the patient that showed features suggestive of multifocal acute infarcts in the right thalamus and bilateral basi-frontal region adjacent to bilateral temporal horns of lateral ventricles (Figure 1).

**Figure 2:**
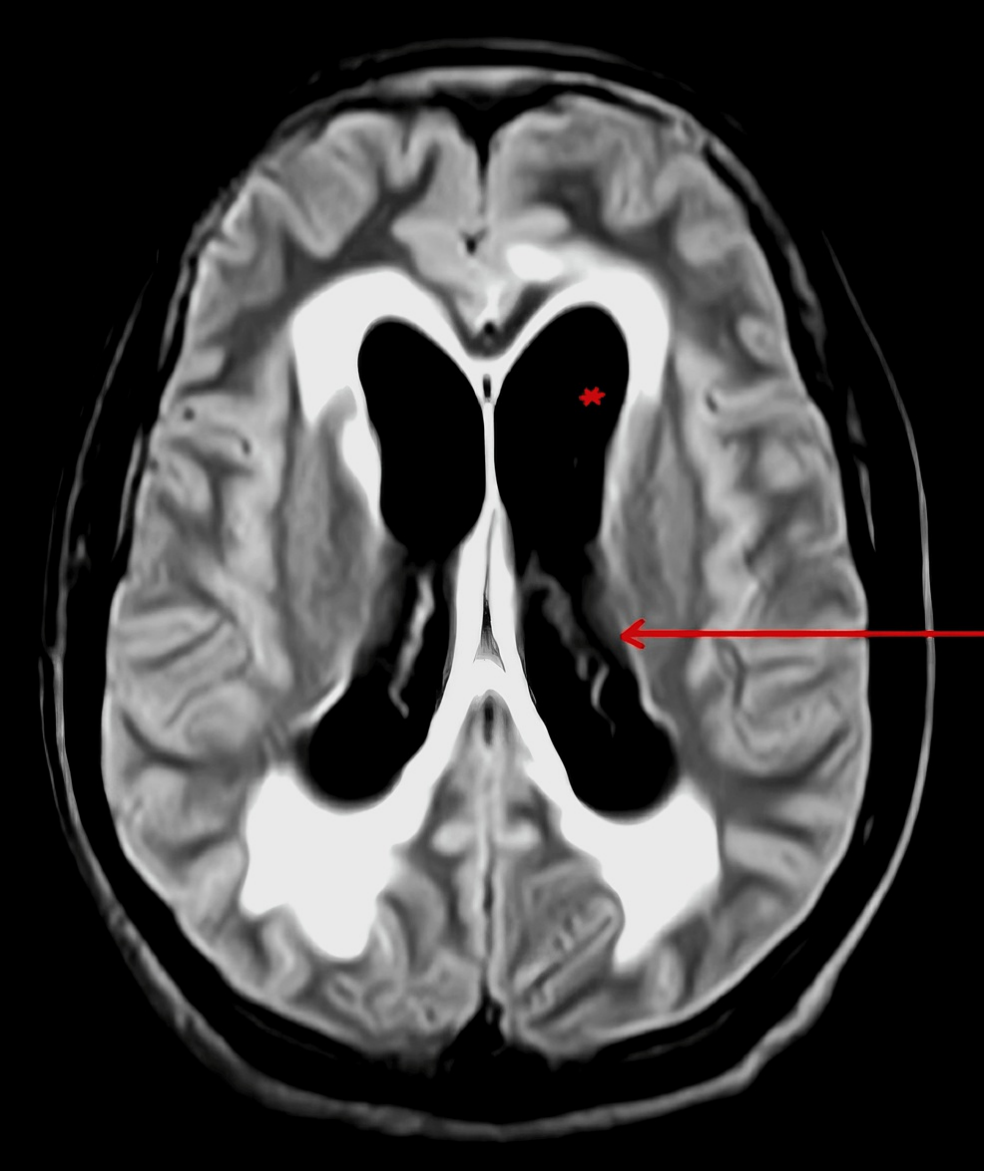
Shows MRI FLAIR image with dilated bilateral ventricle with hyperintensities in periventricular deep white matter. FLAIR- Fluid-attenuated inversion recovery

Consequent MRI done after seven days showed dilatation of the bilateral frontal horn of lateral measuring 18.8 mm, bilateral temporal horn of lateral ventricle measuring 18.4 mm, bilateral posterior horn of lateral ventricle measuring 20 mm, and third ventricle suggestive of obstructive hydrocephalus with periventricular ooze (Figure 2).

**Figure 3:**
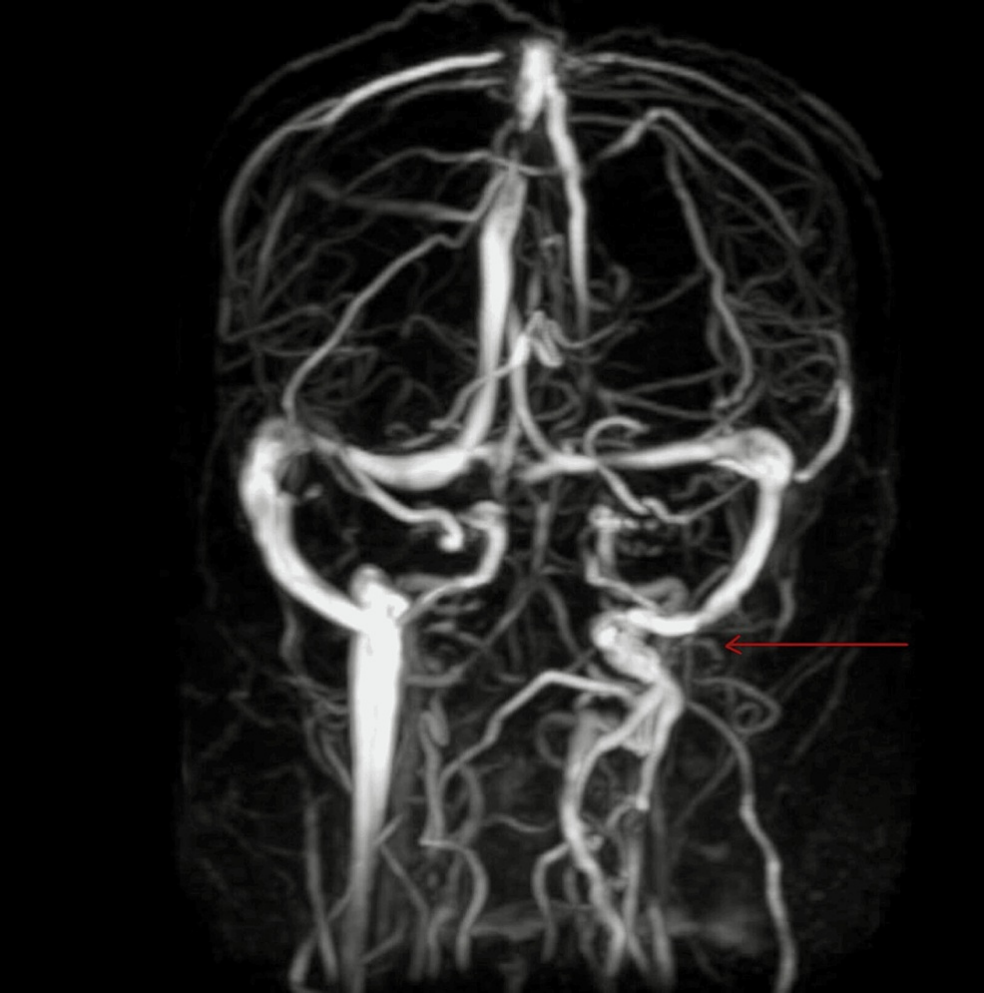
Shows MR venography suggestive of non-visualization of left IJV with multiple collaterals. IJV- internal jugular vein

MR venography showed non-visualisation of left IJV with multiple adjacent collaterals (Figure 3). Bilateral carotid and IJV Doppler were used to rule out chronic thrombus, and a normal bilateral carotid artery was shown. The left IJV shows echogenic walls with increased intimal thickening, peripheral fat stranding, and a few echogenic foci within the lumen, possibly suggesting IJV thrombophlebitis. Mini-BAL (Broncho-Alveolar Lavage) was done and sputum microscopy for acid-fast bacilli and sputum Truenat for multibacillary tuberculosis (MTB) were done and were positive. Sputum Truenat detected 1.9*10^3^ colony-forming units/ml and resistance to rifampicin was not detected. To rule out any further drug resistance, we also sent a second-line line probe assay which did not detect any resistance. On asking relatives, they denied any history of TB or contact.

**Figure 4:**
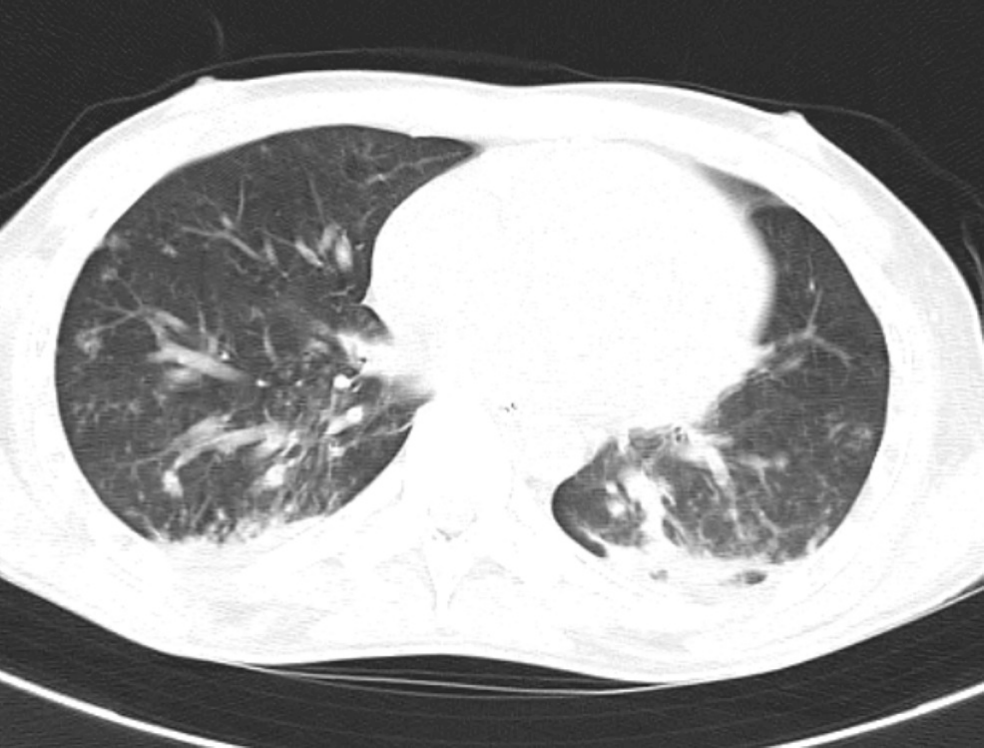
HRCT thorax shows bilateral posterior patchy consolidation. HRCT- High-resolution computed tomography

**Figure 5:**
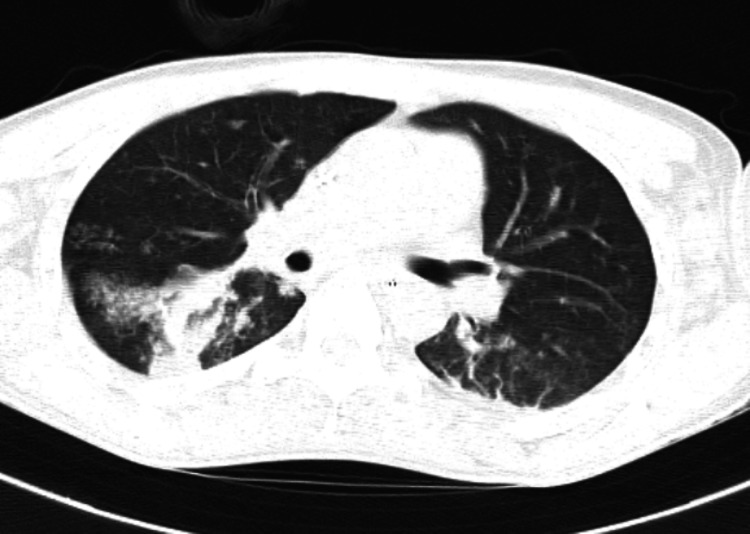
HRCT thorax shows bilateral tree in bud appearance. HRCT- High-resolution computed tomography

HRCT thorax was done and showed subsegmental patchy consolidations in posterior basal segments of both lower lobes, posterior segment of right upper lobe, and superior segment of left lower lobe with small patchy opacities of tree in bud patterns in both lungs with multiple large mediastinal, bilateral cervical, supraclavicular and axillary lymph nodes (Figure 4 and Figure 5).

**Figure 6:**
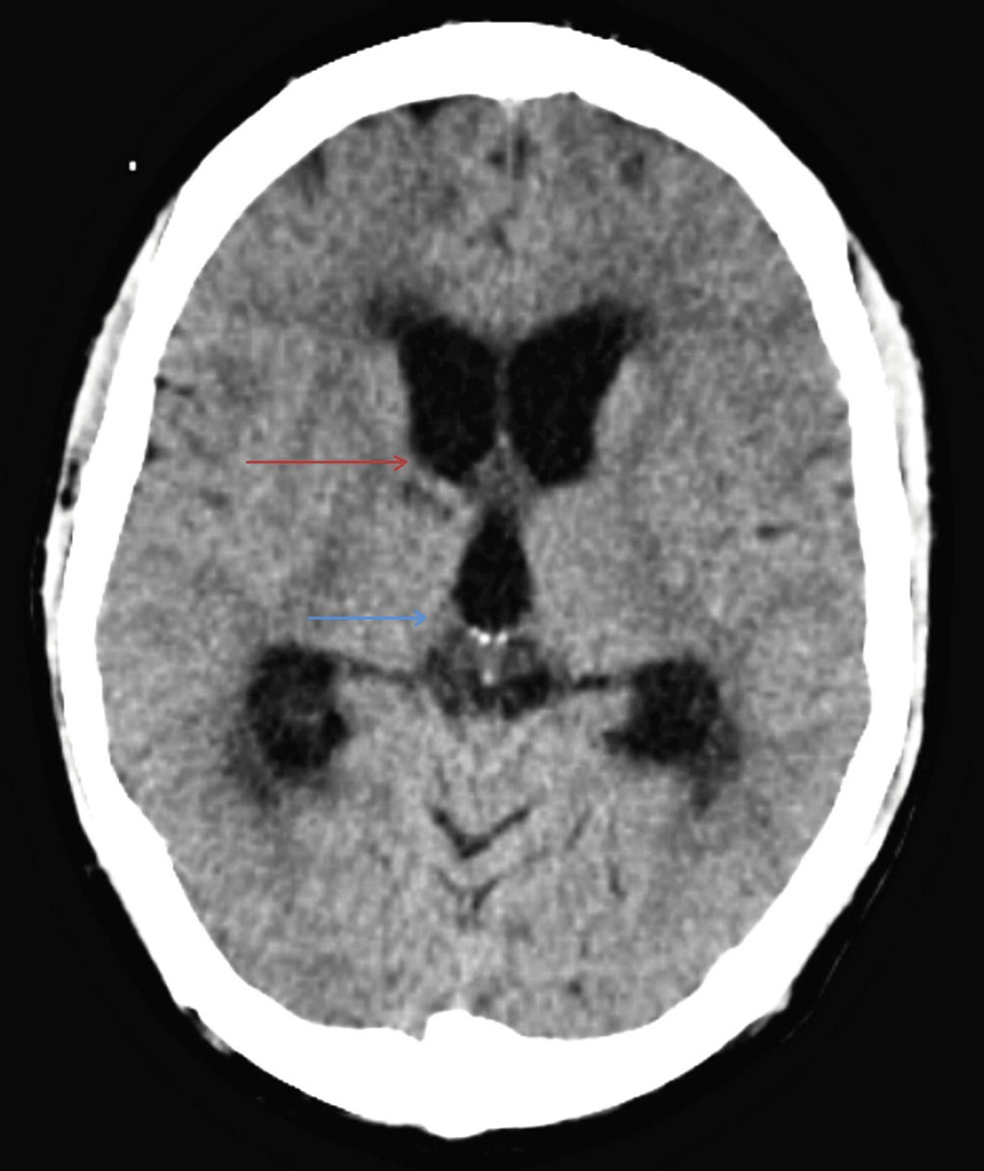
CT brain showing ill-defined hypodense areas in the right caudate nucleus (red arrow) and right thalamus (blue arrow).

Cerebrospinal fluid (CSF) analysis was done, which was positive for adenosine deaminase (ADA), lymphocyte predominant leucocytosis, low glucose, and elevated protein suggestive of TBM (Table 2). A CT of the brain was done, which suggested a subacute infarct in the right head of the caudate nucleus and right thalamus (Figure 6).

**Table 2:** Initial laboratory investigations Sr.- Serum; CSF- Cerebrospinal Fluid; LDH- Lactate dehydrogenase

PARAMETERS	VALUES	NORMAL RANGE
Haemoglobin(gm/dl)	10.1	11-15
Total White Blood Cell Count (per cubic (cu.) mm)	8700	4000-10000
Total Red Cell Count (million per cu .mm)	3.25	4.2-5.4
Platelets (per cu. mm)	191000	140000-440000
Haematocrit (%)	30	36-46
Urea (gm/dl)	23	10-45
Serum (Sr.) Creatinine (mg/dl)	0.7	0.2-1.2
Sr. Sodium (meq/l)	119	135-148
Sr. Potassium (meq/l)	4.0	3.5-5.3
Sr. Magnesium (mg/dl)	1.4	1.8-2.6
Erythrocyte Sedimentation Ratio (ESR)	95	0-15
Serum Glutamyl-Oxaloacetate Transaminase (U/l)	34	11.8-64.8
Serum Glutamyl- Pyruvate Transaminase (U/l)	43	8.5-49.5
Alkaline Phosphatase (U/l)	69	38-126
Sr. Albumin (gm/dl)	2.7	3.2-5.1
Sr. Total Bilirubin (gm/dl)	0.5	0-0.8
International Normalised Ratio	1.18	1.3-1.5
CSF Lactose dehydrogenase (LDH) (I.U./L)	179	200-400
CSF Protein (mg/dl)	823	12-60
CSF pH	7.5	7.4-7.5
CSF Glucose (mg/dl)	22	
Blood Glucose (mg/dl)	156	70-140
Sr. Homocysteine (mmol/L)	2.0	6.6-14.8
Sr. ionized calcium (mEq/L)	4.46	4.4-5.2
Sr. LDH (U/l)	145	140-280

The patient was initially given intravenous (IV) ceftriaxone and vancomycin with an injection of dexamethasone 8 mg 6 hourly; prior to this, CSF was sent for testing. The patient was started on antitubercular therapy with intramuscular streptomycin 750 mg once a day. He was also started on anti-seizure medication IV levetiracetam 500 mg twice a day with a single antiplatelet tablet aspirin 150 mg once a day, injectable low molecular weight heparin 40 mg subcutaneous once daily, and a statin tablet atorvastatin 20 mg once a day at night. Nutritional support was given with total parenteral nutrition (TPN) for three days, after which the patient regained consciousness and was given Ryle’s tube feeding according to his caloric requirement. The patient was weaned off the ventilator over the course of the intensive care unit (ICU) stay and extubated after six days, following which he was put on an oxygen face mask with 4 litres of oxygen.

**Figure 7:**
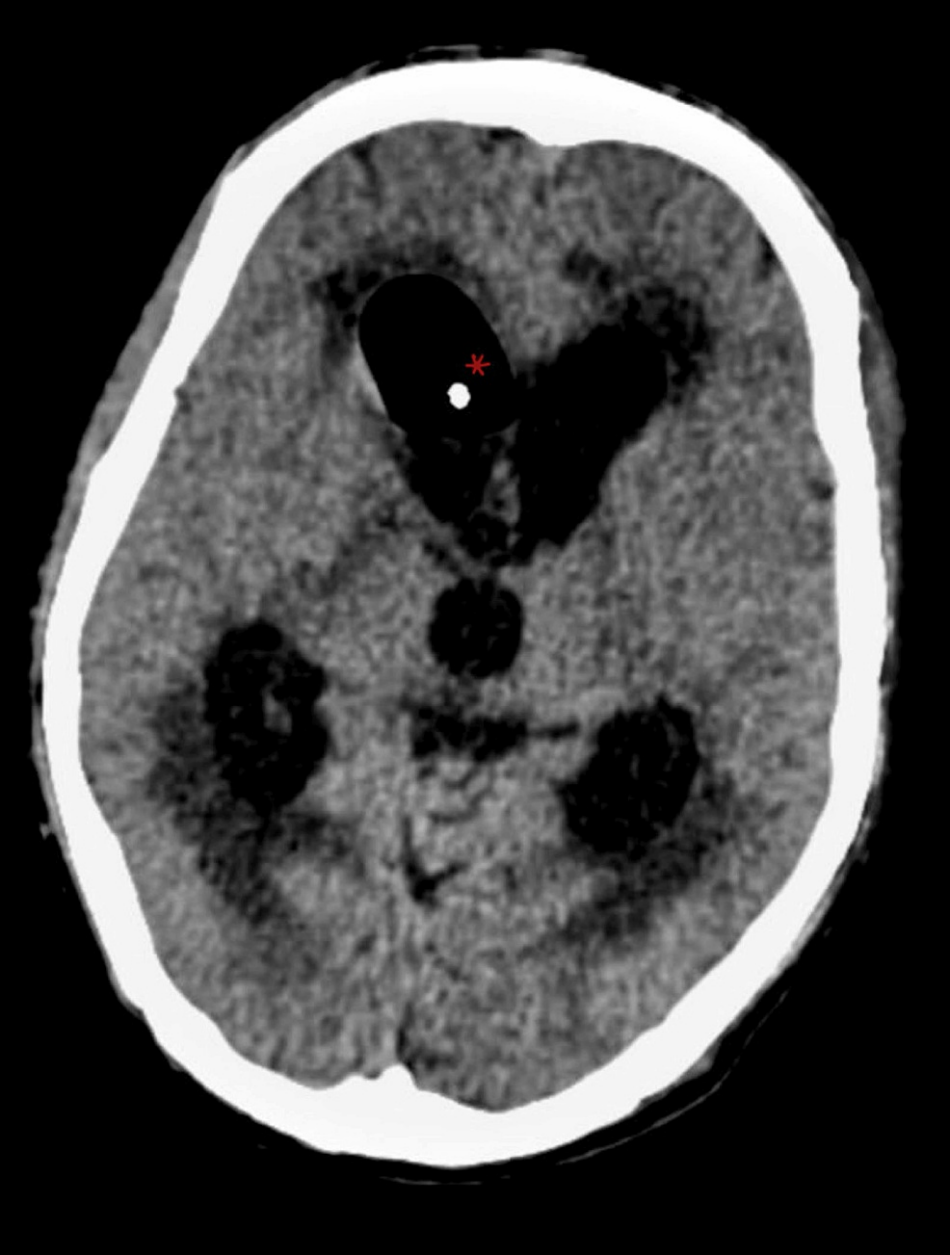
CT Brain image showing dilated bilateral lateral ventricles with periventricular ooze and Ommaya shunt tip in frontal horn of right lateral ventricle.

The neurosurgeon's opinion was taken, and the patient was advised that the Ommaya reservoir and 30 ml CSF were drained intra-op along with daily 10-15 ml CSF drainage for the following two days. CT brain was done before and after Ommaya shunt placement, which suggested a moderate reduction in hydrocephalus with no periventricular leak and multiple foci in bilateral basal ganglia and right thalamus, suggesting subacute infarcts (Figure 6 and Figure 7). Clinical progress was made, and the patient was discharged without incident. In follow-up, the patient remained vitally stable.

**Figure 8:**
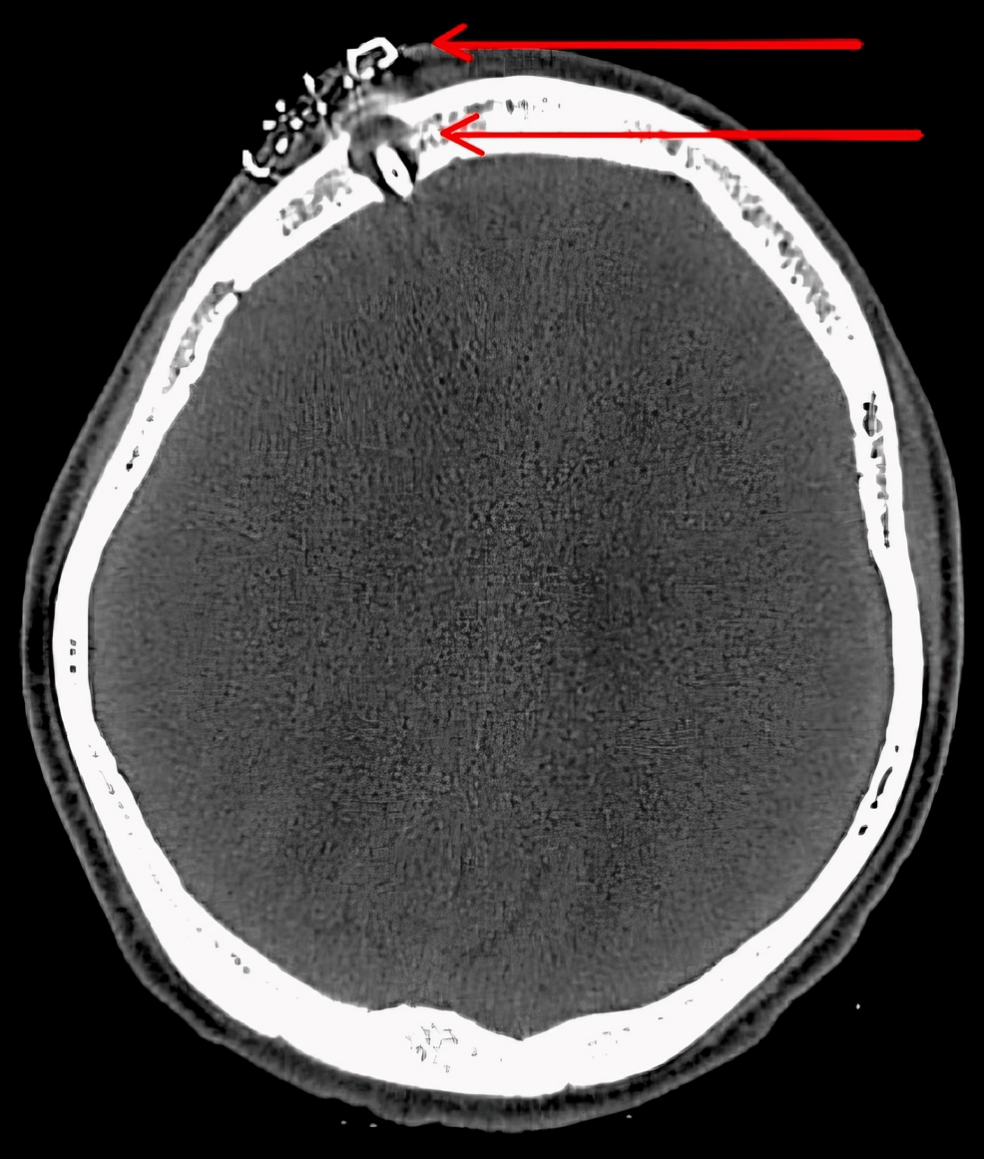
CT brain showing post-operation calvarial defect in right frontal bone showing Ommaya shunt in situ with subcutaneous post-operation sutures.

## Discussion

Lemierre syndrome is an uncommon condition that disproportionately impacts young adults in good health [5]. André Lemierre originally referred to it as an "anaerobic postanginal septicemia" in 1936. Between 1998 and 2001, research in Denmark found that the yearly incidence for adults between the ages of 14 and 24 was 14.4 cases per million. In contrast, the population-wide incidence was 3.6 cases per million [5]. The most common causative microbe for this septic thrombophlebitis is *Fusobacterium necrophorum* [1]. Prior to the discovery of antibiotics, this illness was common and might be fatal in 7 to 15 days [2]. Lemierre's syndrome decreased in frequency as antibiotics became widely used [6,7]. It has become a legend with few recorded instances and a reputation as a "forgotten" sickness that can cause a range of complications and death if not diagnosed promptly [7-9]. The initial lesion in most Lemierre syndrome patients is pharyngitis infecting the peritonsillar region or the palatine tonsils. Dental infections, sinusitis, otitis media, parotitis, and mastoiditis are less frequent main causes of infection [10]. After 1-3 weeks, the infection spreads to the pharyngeal cavity and IJV, which results in septic thrombophlebitis [11].

One reported case even started after blunt damage to the cervical area [12]. Inflammation can also occur in the internal carotid artery inside the carotid sheath [13]. The most typical signs and symptoms of Lemierre's syndrome are sore throat, chest discomfort, neck lump, cervicalgia, difficulty breathing, cough/hemoptysis, otalgia, odontalgia, and abdominal discomfort. Because sternocleidomastoid muscle pain is often unilateral, Lemierre's condition may worsen when the head is turned away from the afflicted side [11]. Chest discomfort and pulmonary symptoms are the most reliable markers of metastatic illness, as the primary location for metastatic illness is the lungs. Lemierre's syndrome physical exam results vary and are influenced by the degree of metastatic illness. Fever, pharyngitis/peritonsillar abscess, neck lump/tenderness, anterior cervical lymphadenopathy, trismus, septic arthritis (usually hip or knee), jaundice/hepatomegaly, palsy of cranial nerve X, XI, and XII, particularly, and shock are the most frequent physical exam findings associated with Lemierre's syndrome.

The appearance and repetition several days after the onset of a sore throat (and particularly of a tonsillar abscess) of several pyrexial attacks with an initial rigour, or still more certainly the occurrence of pulmonary infarcts and arthritic manifestations, constitute a syndrome so characteristic that mistake is almost impossible," Lemierre wrote in his original case series [14]. This would have been the case in the days before antibiotics; the majority of doctors working today probably won't come across a patient with this illness.

Getting samples for culture and radiography of the neck and chest are part of the diagnostic assessment. Additional assessments for problems should also be customized based on the results of a physical exam and history. When IJV thrombus is visible on imaging studies, and F. necrophorum or other associated pathogens are detected in culture data, Lemierre syndrome can be diagnosed. Microorganisms from throat, blood, and metastatic illnesses can all be isolated. A contrast-enhanced CT scan of the neck and chest facilitates the evaluation of the IJV for filling abnormalities or thrombus. Assessing for pulmonary involvement, including lung emboli and abscesses, is enabled by this imaging [15]. To assess for neurologic sequelae, patients with headaches, meningeal symptoms, or neurologic impairments should ideally have magnetic resonance imaging. Timely identification is essential for suspected Lemierre's syndrome to prevent sepsis and slow down the advancement of the illness; however, delayed diagnosis is common due to the condition's relatively mild nature and limited understanding [16].

In our case, we ruled out other differential diagnoses, the first being right-sided endocarditis. It should come as no surprise that a considerable portion of patients diagnosed with Lemierre's condition first get treatment for either right-sided staphylococcal endocarditis or pneumonia [17,18]. Patients who have persistent bacteremia following 72 hours of proper treatment and a catheter-related bloodstream infection should be suspected of having catheter-associated septic thrombophlebitis. In addition to potential initial signs of the infection, sequelae might include subsequent pneumonia and septic pulmonary emboli. This was ruled out because our patient had never been hospitalized before.

The determination of Lemierre's syndrome diagnosis may only be finalized within a suitable clinical context. 90% of the cases in Lemierre's initial case series from the pre-antibiotic period succumbed [14]. Research from the contemporary age indicates that death rates range from 0% to 18% [5,11,17].

## Conclusions

Contemporary throat infection, evidence of thrombosis in the IJV, either clinical or radiographic imaging, and anaerobic bacteremia predominantly implicating *F. necrophorum* are Lemierre's syndrome's hallmarks, primarily impacting young people in otherwise good health.

Anaerobic bacterial growth on blood culture and imaging studies are commonly used to confirm the diagnosis of IJV thrombophlebitis. The mainstay of treatment is extended antibiotic therapy, occasionally paired with anticoagulation. Seldom-occurring causes of IJV thrombosis include tuberculous septic thrombophlebitis and tuberculous cervical lymphadenopathy. Early diagnosis using CT and/or USG is necessary to prevent future problems and to start therapy on time.

Acute ischemic stroke affects around 25% of young individuals with TBM, and this condition may be linked to worse clinical outcomes. We present an unusual case of meningitis, acute ischemic stroke, obstructive hydrocephalus, and Lemierre's syndrome associated with TB. In this instance, the unique manifestation and undetected risk factor of Lemirre's syndrome emphasize how crucial it is to take the relationship between primary pulmonary tuberculosis infections and vasculitis into account. Through this case, we wish to raise awareness among physicians regarding the possibility of Lemierre’s syndrome in TB and improved prognosis with early identification and treatment.
